# Continual conscious bioluminescent imaging in freely moving somatotransgenic mice

**DOI:** 10.1038/s41598-017-06696-w

**Published:** 2017-07-25

**Authors:** Rajvinder Karda, Dany P. Perocheau, Natalie Suff, Joanne Ng, Juliette M. K. M. Delhove, Suzanne M. K. Buckley, Samantha Richards, John R. Counsell, Henrik Hagberg, Mark R. Johnson, Tristan R. McKay, Simon N. Waddington

**Affiliations:** 1 Gene Transfer Technology Group, Institute for Women’s Health, University College London, London, UK; 2 Institute of Child Health, University College London, London, UK; 3 Department of Perinatal Imaging & Health, King’s College London, London, UK; 4 Department of Surgery and Cancer, Imperial College London, London, UK; 5 Centre for Biomedicine, Manchester Metropolitan University, Manchester, UK

## Abstract

Luciferase bioimaging in living animals is increasingly being applied in many fields of biomedical research. Rodent imaging usually involves anaesthetising the animal during data capture, however, the biological consequences of anaesthesia have been largely overlooked. We have evaluated luciferase bioimaging in conscious, unrestrained mice after neonatal intracranial or intravascular administration of lentiviral, luciferase reporter cassettes (biosensors); we present real-time analyses from the first day of life to adulthood. Anaesthetics have been shown to exert both neurotoxic and neuroprotective effects during development and in models of brain injury. Mice subjected to bioimaging after neonatal intracranial or intravascular administration of biosensors, targeting the brain and liver retrospectively showed no significant difference in luciferase expression when conscious or unconscious throughout development. We applied conscious bioimaging to the assessment of NFκB and STAT3 transcription factor activated reporters during the earliest stages of development in living, unrestrained pups. Our data showed unique longitudinal activities for NFκB and STAT3 in the brain of conscious mice. Conscious bioimaging was applied to a neonatal mouse model of cerebral palsy (Hypoxic-Ischaemic Encephalopathy). Imaging of NFκB reporter before and after surgery showed a significant increase in luciferase expression, coinciding with secondary energy failure, in lesioned mice compared to controls.

## Introduction

The use of germline transgenic and somatic transgenic luciferase bioimaging in mice is well established in the study of cell reprogramming^1^, cell engraftment^2^, tumour tracking^3^, gene therapy^4^ and disease pathogenesis^5^. We have previously described a library of lentiviral transcription factor activated reporter (TFAR) vectors carrying the luciferase transgene under the transcriptional control of a minimal promoter and serial transcription factor binding elements. Targeted perinatal administration enables real-time biosensing of nuclear factor kappa B (NFκB) activity in the brains, liver and lungs of neonatal rodents^6^. This technology exploits the fact that gene delivery to neonatal rodents achieves immune tolerance to the transgenic protein^7^, in this case being firefly luciferase.

Cell signalling is often stimulated by extrinsic stimuli. Several studies have shown that isoflurane anaesthetic can disrupt physiological and pathological processes, notably in the brain. In mice, isoflurane reduces primary neuronal axon outgrowth and partially inhibits the ability of primary mouse astrocytes to support neuronal growth^8^. It has been shown to alter CLOCK gene expression, thereby affecting circadian rhythms^9^. Similar observations of alterations to circadian rhythms^10, 11^, increased basal ganglia GABAergic activity within the basolateral amygdala^12^ and cognitive impairment^13^ have been associated with isoflurane exposure. Additionally, isoflurane exposure has shown to cause microglia activation, cell death and disrupt expression of genes associated with cognitive function and neurodevelopment in newborn male piglet brains^14^. Neurological defects have also been associated with other anaesthetic agents. For instance, exposure to propofol induces neuroapoptosis in a developing rodent brain^15^ and sevoflurane causes cell death in the neonatal mouse brain leading to abnormalities within their social behaviour and induced learning difficulties^16^.

Whole-body bioluminescence imaging of light-producing germline or somatic transgenic rodents is routinely performed under general anaesthesia to prevent movement and, therefore, to localise sites of expression as regions of interest for photon capture. Since lentivirus targeted somatic transgenesis achieves organ-restricted reporter gene expression, rodents could remain conscious and unrestrained during imaging. We compared conscious and unconscious bioimaging in the brains and livers of mice using a constitutively active luciferase reporter and two TFAR during post-natal development until adulthood. We show that conscious bioimaging of brain and liver expressing somatotransgenic mice is feasible and comparable to unconscious imaging. Imaging from visceral organs such as the liver is made possible by using simple light reflection principles. Moreover, we applied these methods of conscious bioimaging of the brain in a neonatal model of Hypoxic Ischemic Encephalopathy (HIE). The rodent model of HIE is well established^17^ and replicates the pathology and physiology of perinatal asphyxia, which often results in neurological disorders such as cerebral palsy and epilepsy^18^. We present data monitoring inflammation in a non-invasive manner by conscious bioimaging. Crucially, it is a substantial refinement of animal welfare and thus encouraging 3Rs principles in state-of-the-art whole-body imaging modalities.

## Results

### Establishing somatotransgenic technology within the mouse CNS and assessing the effects of isoflurane on bioimaging

Here we report, for the first time, that whole-body bioluminescence imaging can be conducted on conscious mice from birth onwards, using organ-targeted biosensors. A lentiviral vector containing the constitutive SFFV viral LTR/promoter driving an codon-optimised luciferase transgene linked to an enhanced GFP by a bicistronic linker was pseudotyped with wide tropism VSV-G envelope protein^19^.

**Figure 1:**
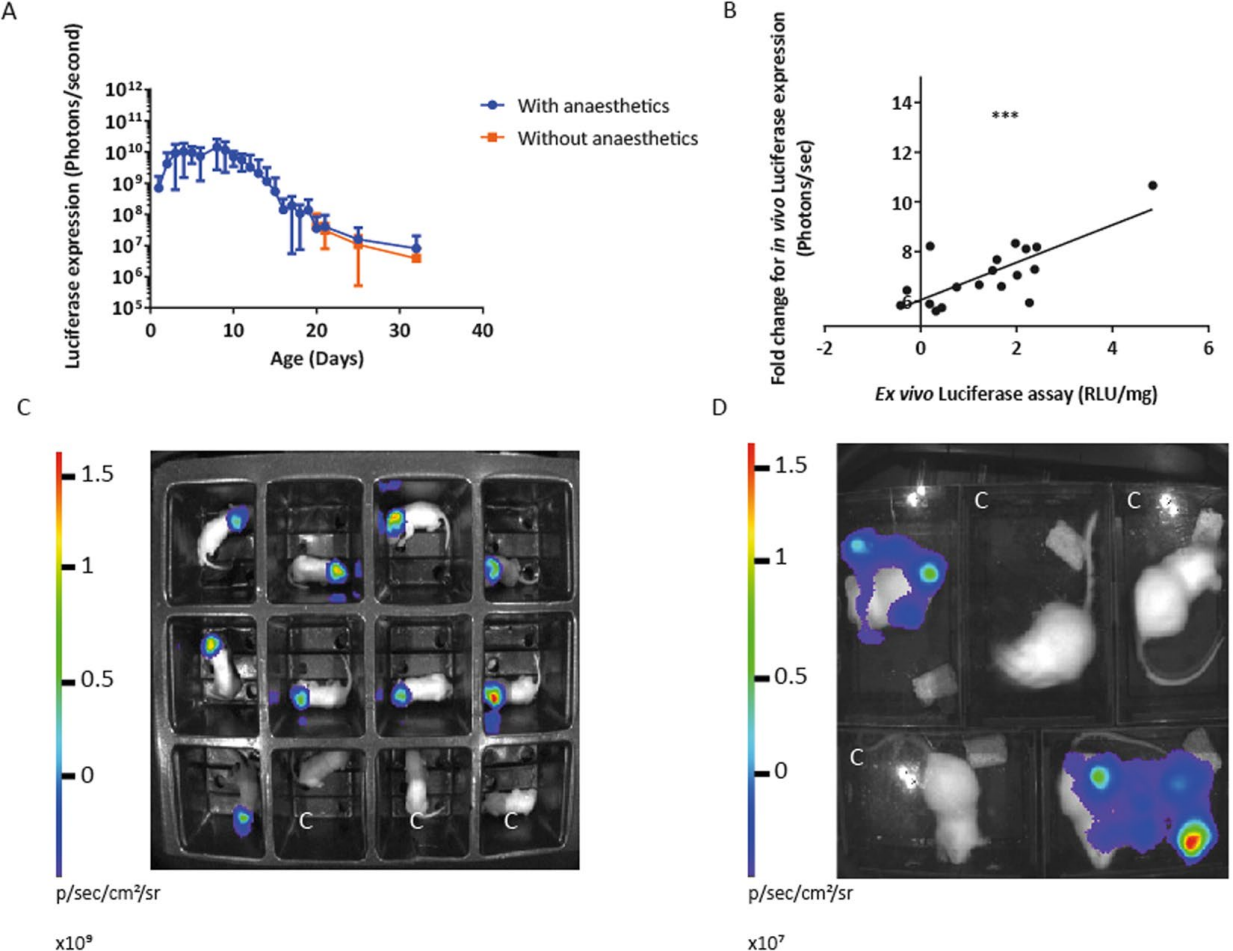
Intracranial neonatal administration of VSV-G SFFV lentiviral biosensor, with mean bioluminescence quantified from anaesthetised and non-anaesthetised mice. Mice received the VSV-G pseudotyped lentivirus vector containing the constitutive SFFV promoter, at birth via intracranial injections (n = 9). They were imaged daily, without anaesthetic, until 21 days old. (**A**) Mean bioluminescence was quantified. The mice were then allocated randomly into two groups and whole-body bioluminescence imaging was undertaken either with or without isoflurane anaesthesia. There was no significant difference in luciferase expression between the two groups, (mean +/− SD, P = 0.657). (**B**) New born mice received an intracranial injection of VSV-G SFFV lentiviral vector. Over the course of development mice were bioimaged and brain tissue from three mice was collected at weekly time points for *ex vivo* luminometry (n = 3). A strong positive correlation was observed between the fold change of *in vivo* luciferase expression at the time of euthanasia and *ex vivo* luciferase expression, P = 0.0003, R^2^ = 0.5690. (**C**) Imaging of neonatal mice and (**D**) adult mice without anaesthetic, mice marked with C are control mice which have not received vector. The region of interest (ROI) used to quantify conscious neonatal mice in each enclosure, was 5 cm by 5 cm. Once the mice had reached adult hood the ROI for both conscious and unconscious mice was 9 cm by 12.5 cm. The exposure time for each bioluminescent image quantified was 1 minute.

Vector of 1×10^9^ viral particles/ml was administered to newborn (P0/1) outbred CD1 mice by intracranial injection. We have shown that neonatal intracranial administration of lentivirus vector does not induce an immune response and scar the CNS tissue, compared to adult intracranial injections (Supplementary Figure 1), which agree with previous work^20^. After P0/1 lentiviral administration conscious mice were imaged (without anaesthetic) every 48hrs until 21 days old and their bioluminescence quantified (Fig. 1A). To measure the effects of isoflurane on the luciferase expression, from day 21 we randomly allocated mice to one of two groups. One group underwent whole-body bioluminescence imaging while conscious (no isoflurane) and breathing atmospheric air whereas the other half-received isoflurane anaesthesia and 100% oxygen during imaging. There was no significant difference in luciferase expression between groups imaged with or without anaesthesia post-weaning (Fig. 1A) showing that unrestrained conscious bioimaging is at least equally effective as in anesthetised mice.

We sought to confirm whether *in vivo* luciferase expression was an accurate representation of luciferase expression in isolated tissue. VSV-G SFFV lentiviral vector of (1 × 10^9^ vector particles/ml) was administered to newborn (P0/1) CD1 mice by intracranial injection (n = 18). Over the course of development whole-body bioluminescence imaging was conducted at weekly time points, brain tissue was collected from three mice for *ex vivo* luminometry. There was a strong positive correlation between the fold change of luciferase expression *in vivo* and *ex vivo* luciferase expression (P = 0.0003, R^2^ = 0.569) (Fig. 1B) proving that conscious bioimaging is a faithful representation of tissue specific luciferase activity.

### Conscious bioimaging achieved within the liver

Having demonstrated the utility of conscious imaging of brain-targeted lentivirus, we tested whether it was feasible to perform conscious imaging in other discrete tissues. We selected a liver-targeting system since we have shown this to be amenable to bioluminescent imaging in anaesthetised mice^6^. Moreover, the liver is a major site of metabolism of anaesthetics through the activity of Cytochrome P450 oxidases^21^.

**Figure 2:**
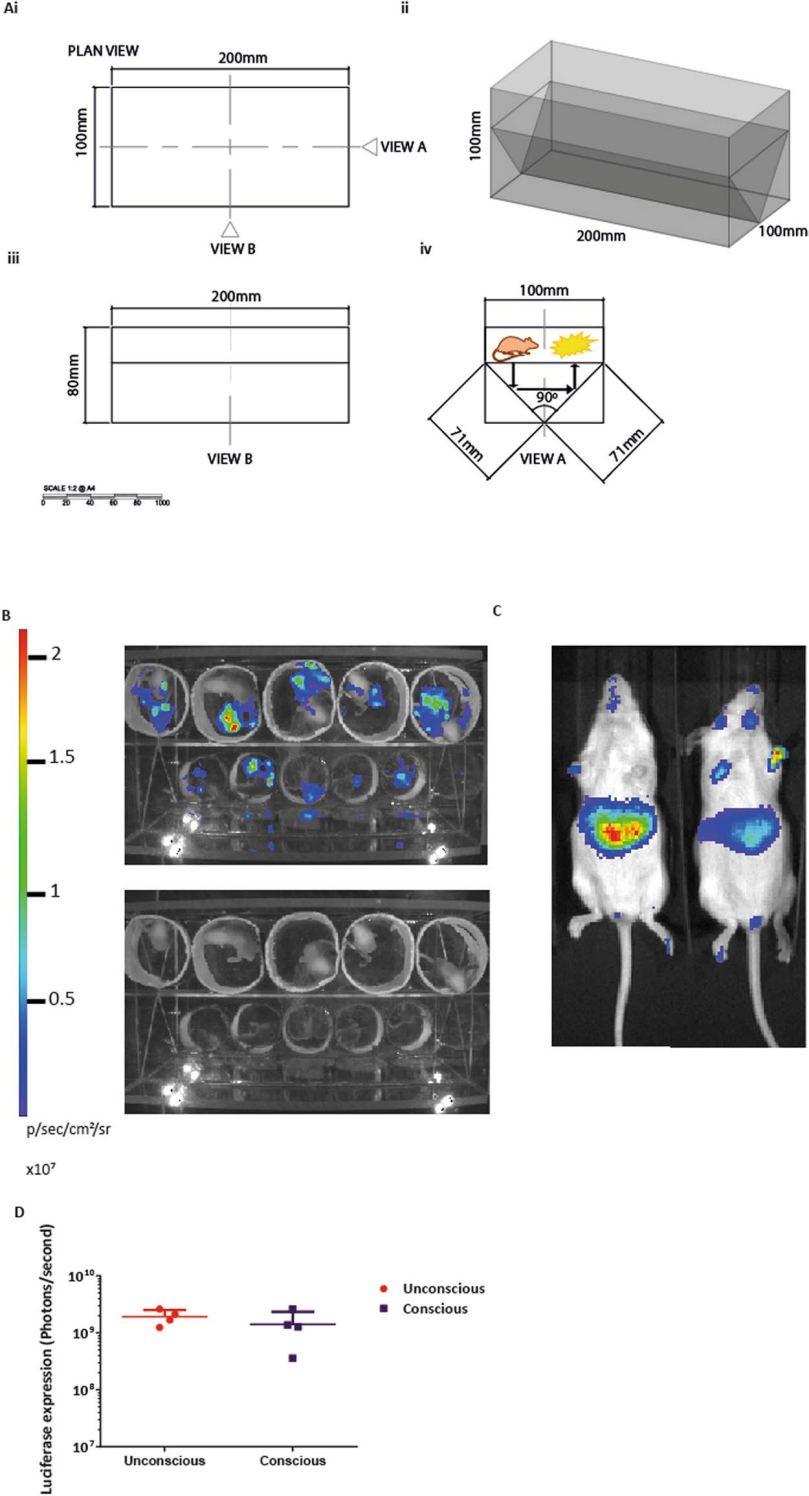
Whole-body bioluminescence imaging of conscious mice which have received a neonatal intravenous injection of VSV-G SFFV biosensor. **(Ai)** Plan view of the chamber, the scale bar represents millimetres (mm). **(Aii)** A 3D model with measurements (mm) of the Perspex box. **(Aiii)** A side-view B represents the length and depth of the chamber. **(Aiv)** Elevation view A shows the 90 degrees angle of the mirrors to construct a periscopic chamber to permit simultaneous collection of light emission from both ventral and dorsal surfaces, shown by the arrows. Mice were administered with VSV-G SFFV lentiviral vector intravenously at birth (n = 8). **(B)** Mice were imaged while conscious using the Perspex chamber, 5 at a time as neonates. The top image shows the whole-body bioluminescence and the lower image shows the mice in the chamber. The luciferase expression obtained from the conscious mice in the Perspex chamber was quantified by adding the expression profiles from the two planes. **(C)** Post weaning the mice were randomly split into two groups, where half of the mice were imaged unconsciously (not in the Perspex chamber). **(D)** There was no significant difference of luciferase expression between the conscious and unconscious mice, P = 0.657, mean +/− SD.

A problem specific to imaging of ventrally-situated organs in conscious mice is that photons are normally captured from above and, therefore, signal may be substantially attenuated as it passes through the animal. We constructed a periscopic chamber to permit simultaneous collection of light emission from both ventral and dorsal surfaces (Fig. 2A).

Neonatal mice received VSV-G pseudotyped SFFV-FLuc/GFP (biosensor) lentivirus intravenously at P1. The luciferase expression from these mice was quantified by whole-body bioluminescence imaging in conscious mice until P17. The mice were imaged while conscious by placing them in the acrylic periscopic box which allowed the light emissions to be projected upwards and thus captured by the CCD camera (Fig. 2A,B). Half of the mice received anaesthetics and the other half were unrestrained and conscious (Fig. 2B,C). Again, there was no significant difference in the luciferase expression between groups proving that unrestrained conscious mice can be subjected to liver-specific luciferase bioimaging with equal efficacy to anaesthetised mice (Fig. 2D; P = 0.657).

### Somatotransgenesis of the CNS achieved with the use of other biosensors

**Figure 3:**
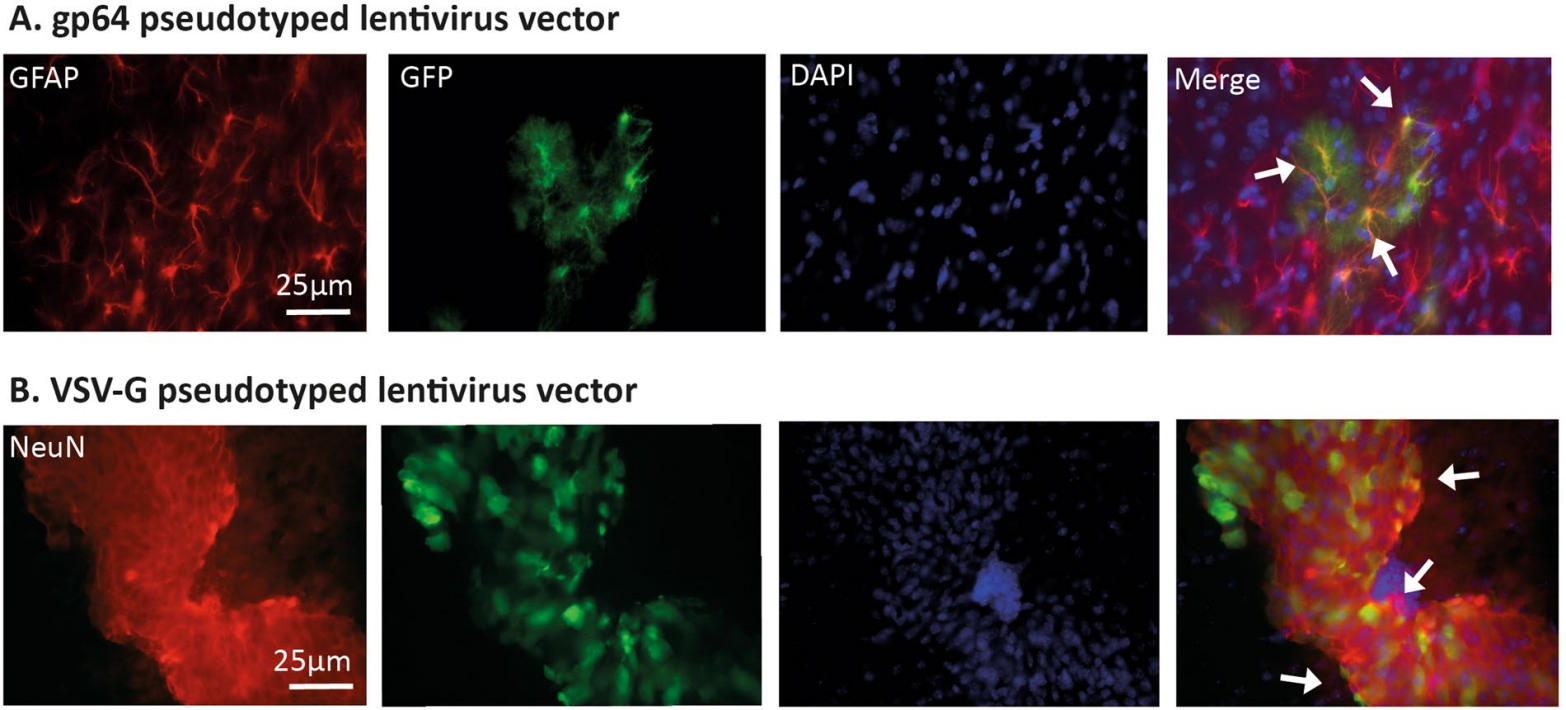
Astrocytic targeting with gp64﻿ pseudotyped lentivirus vector and neuronal targeting with VSV-G pseudotyped lentivirus vector. Brain sections from injected mice were used to determine the cell types expressing GFP after neonatal intracranial injections of gp64﻿ or VSV-G SFFV lentivirus vector. (**A**) GFP with fibrotic GFAP astrocytic signals were co-localised. This is shown with the white arrows in the merge column. (**B**) GFP co-localised with NeuN in the CNS of mice which received the VSV-G SFFV vector (white arrows). All the images were taken at ×40 magnification.

Following our demonstration that neonatal lentiviral vector administration is safe and efficient, and that continual *in vivo* bioimaging is effective in conscious mice, we sought to determine whether we could monitor signalling pathways in different cell populations using different lentivirus vector pseudotypes. Neonatal mice received either VSV-G or gp64 pseudotyped SFFV lentiviral vectors via intracranial injections. The brains of the mice were harvested at 35 days of age and immunofluorescence was conducted on the brain sections. The results showed that lentivirus vectors pseudotyped with either VSV-G or gp64, target neuronal or astrocytic cells retrospectively after a single neonatal intracranial injection (Fig. 3).

We therefore sought to investigate the expression profile of NFκB and STAT3 signalling pathways in two different cell populations within the developing brain of conscious mice. NFκB and STAT3 are transcription factors, which regulate genes associated with cell stress, inflammation and apoptosis and have shown to be involved in pathological processes following excitotoxic brain injury. Early activation of these transcription factors in astrocytes may result in astrogliosis and the release of signalling molecules which may consequently lead to activation of NFκB and STAT3 in microglia^22^.

**Figure 4:**
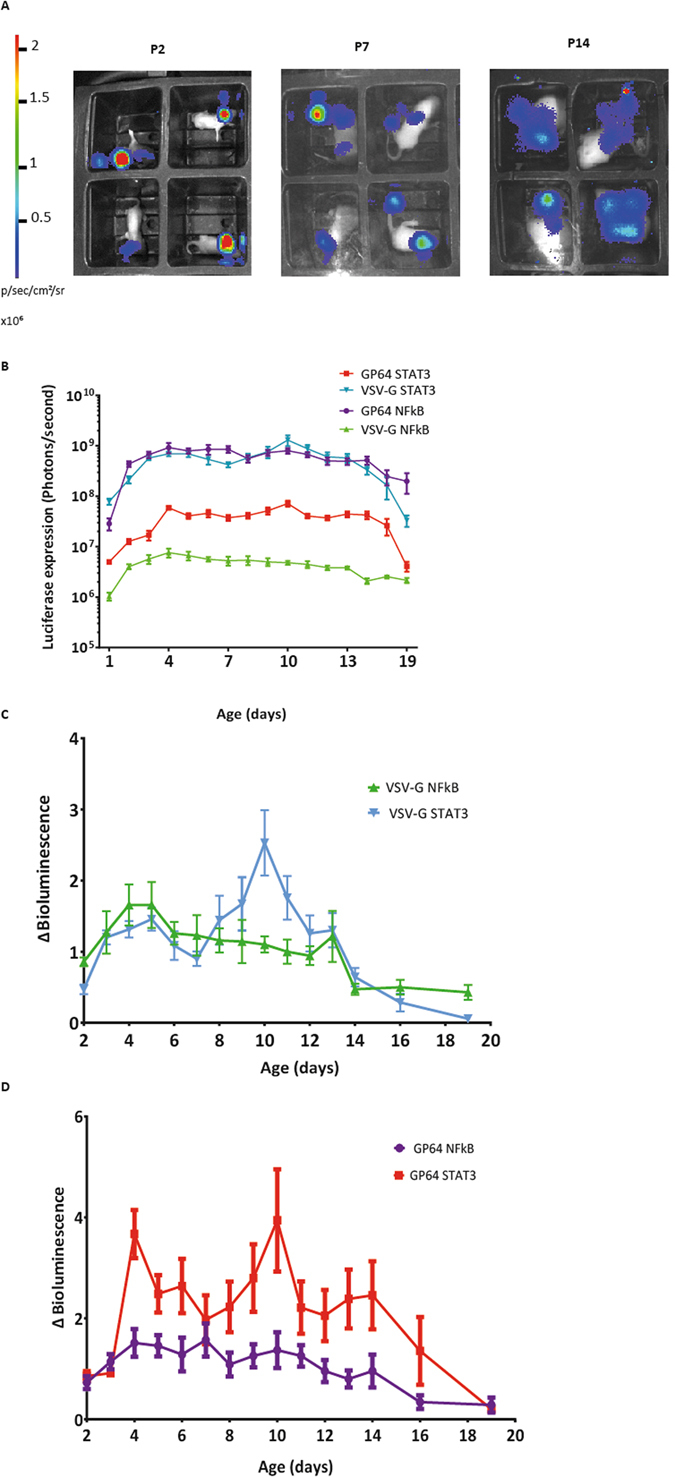
Whole-body conscious bioluminescence imaging of mice after VSV-G or gp64 pseudotyped NFκB or STAT3 lentiviral vector biosensor. (**A**) Representative images are shown at 2, 7 and 14 days of age. The ROI used were 5 cm by 5 cm and exposure time was 1 minute. (**B**) Conscious whole-body bioluminescence imaging of the mice was undertaken daily until P21 (n = 6) for each biosensor. (**C**) A significant peak in luciferase fold change was observed at P10 of development in mice receiving VSV-G STAT3, (mean +/− SD, *P* < 0.001). (**D**) A significant peak was also observed in the gp64 STAT3 mice at P10 of development, (mean +/− SD, *P* < 0.001).

Mice received an intracranial injection of either VSV-G or gp64 pseudotyped lentiviral vector containing the NFκB or STAT3 biosensor (vectors titre matched, 1 × 10^9^ vector particles per ml). These mice were imaged daily without anaesthesia until P14 and then every 48 hours until P19 (Fig. 4A,B). A fold change of raw luciferase expression was calculated and a peak of luciferase expression at P10 of development was observed in both groups of mice which received VSV-G and gp64-pseudotyped STAT3 lentiviral vector, respectively, but no peak was observed in mice which received the NFκB biosensor delivered by either vector pseudotype, P < 0.001 (Fig. 4C,D). These data are in agreement with previous data showing an increase in STAT3 expression at P10 of development in hypothalamic neurons^23^.

### Monitoring the changes of luciferase expression with the induction of HIE

Following our observation that VSV-G pseudotyped lentiviral vector targets neuronal cells in the mouse CNS after neonatal intracranial administration, we sought to interrogate the expression of neuronal NFκB after acute brain injury. The HIE model has been routinely induced on day 7 post-natal C57BL/6 J mice^24^; therefore we first sought to establish the model in the CD1 mouse strain. Following carotid artery ligation, mice were subject to increasing durations of hypoxia. This revealed that 90 minutes of hypoxia produced a variable model where the CNS damage was blind marked and ranged from 1 as normal to 4 as severe (Supplementary Figure 2).

**Figure 5:**
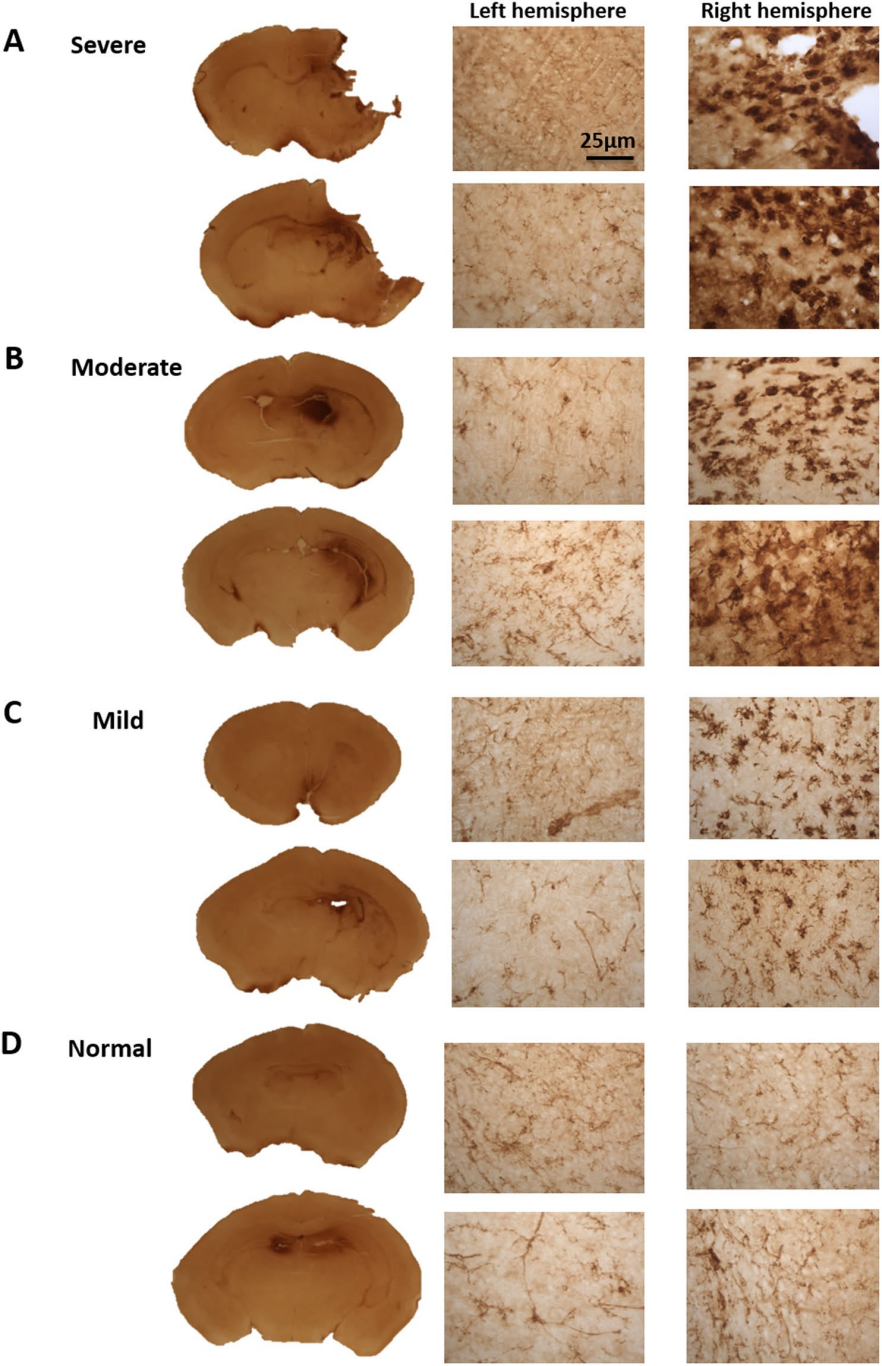
Reactive microglia immunohistochemistry on HIE brains harvested 48 hours after surgery. Reactive microglia was detected by conducting a CD68 immunohistochemistry on the variable HIE brains. (**A**) Severe brains showed reactive microglia within the cortex and the hippocampus of the right lesioned hemisphere. CD68 positive cells were also found within the (**B**) moderate and (**C**) mild right hemisphere. Whereas CD68 expression was, absent from the (**D**) normal brain. The brain section images were taken at ×5 magnification and the left and right hemisphere images were taken at ×40 magnification.

**Figure 6:**
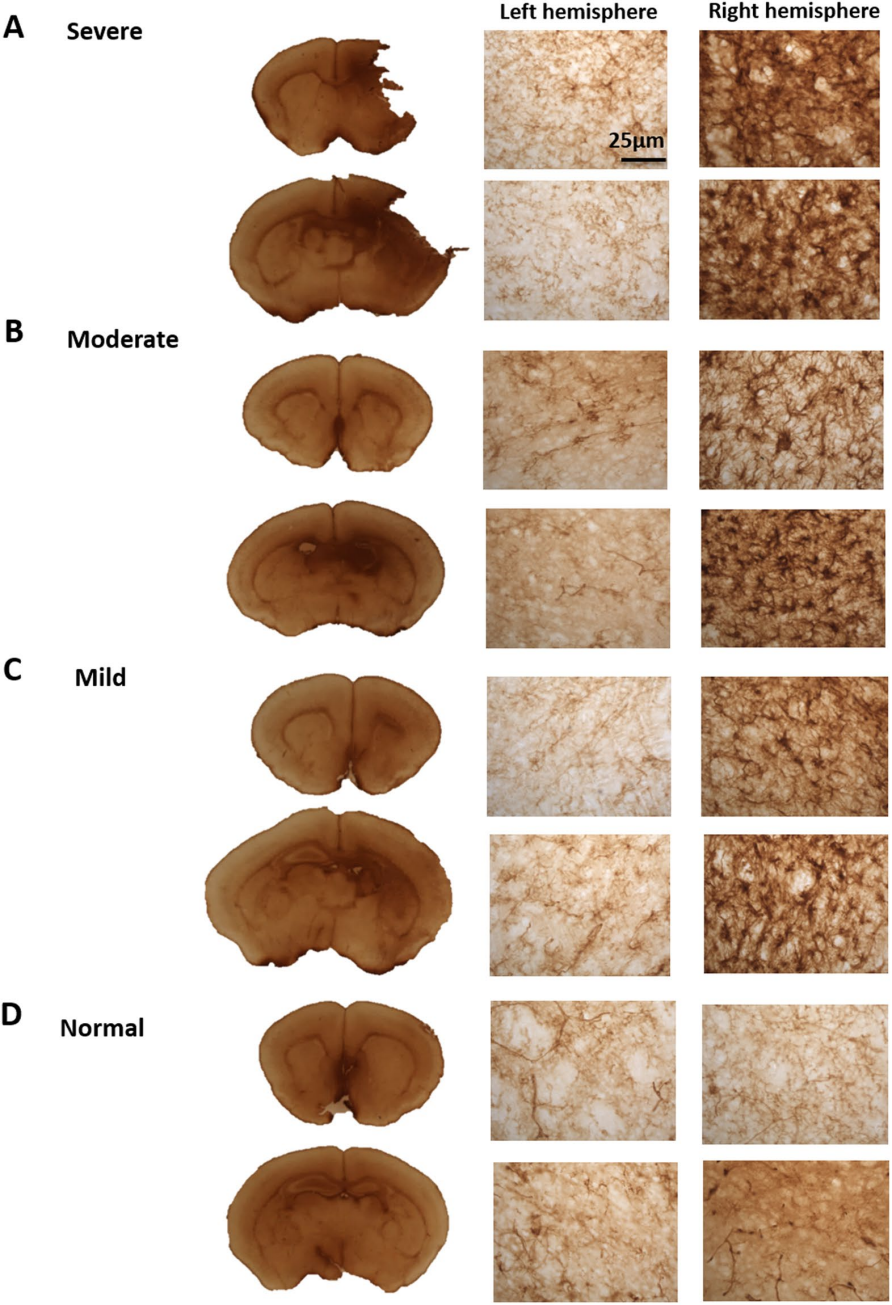
Astrogliosis detection on HIE brains harvested 48 hours after surgery. Astrogliosis was detected by conducting GFAP immunohistochemistry on the variable HIE brains. (**A**) Severe brains showed astrogliosis within the cortex and the hippocampus of the lesioned right hemisphere. GFAP positive cells were also found within the (**B**) moderate and (**C**) mild right hemisphere. Whereas the expression was absent from the normal brain (**D**). The brain section images were taken at ×5 magnification and the left and right hemisphere images were taken at ×40 magnification.

**Figure 7:**
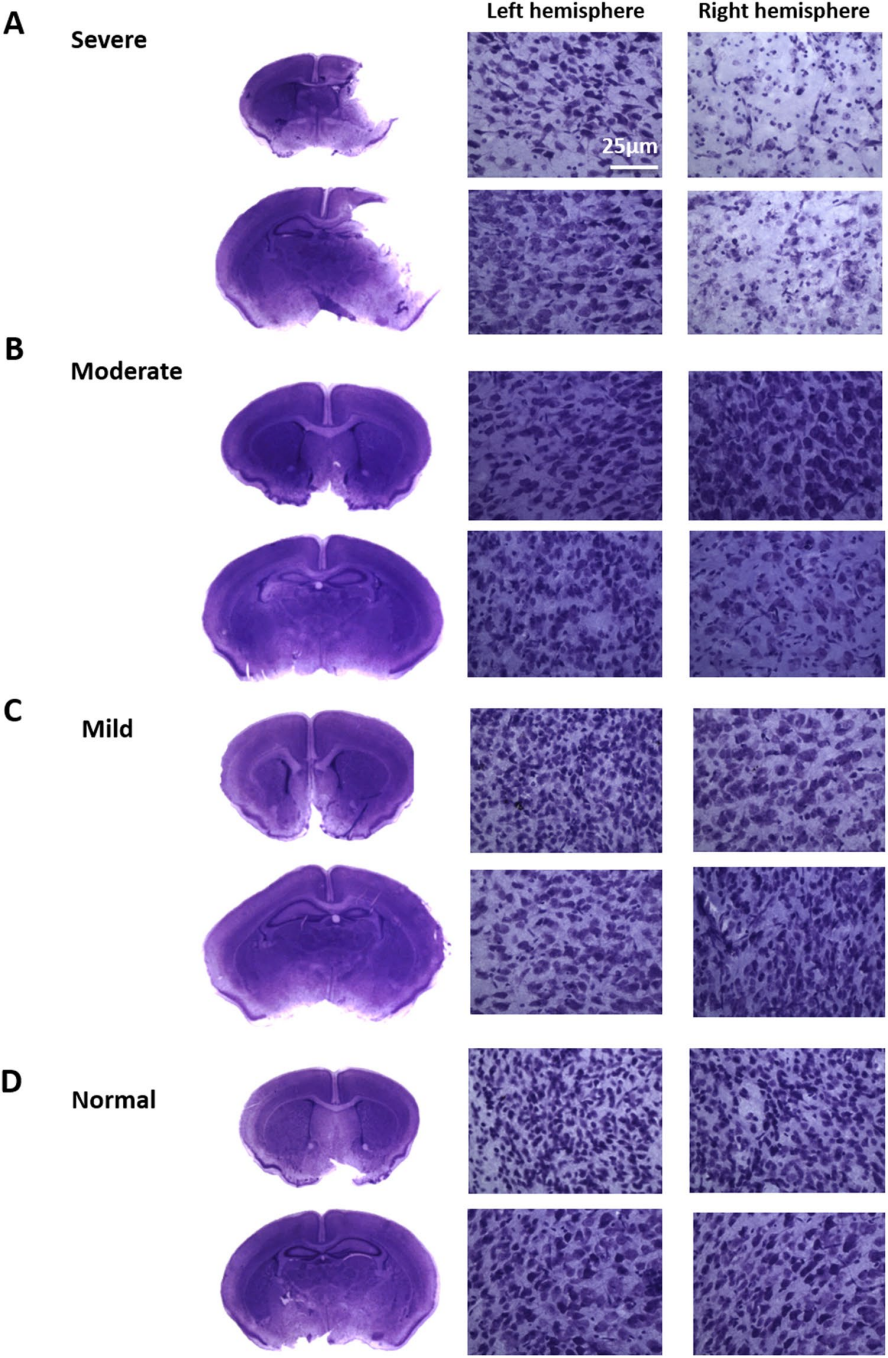
Nissl stain on HIE brains harvested 48 hours after surgery. Nissl stain represents rough endoplasmic reticulum, ribosomal RNA and DNA. Nissl bodies are seen within Neuronal cell bodies, however disappear in response to ischemia due to neuronal cell death. (**A**) Brains showed neuronal cell death within the cortex and hippocampus of the lesioned right hemisphere. Nissl bodies were somewhat absent within the (**B**) moderate and (**C**) mild right hemispheres. Whereas Nissl bodies were present within the (**D**) normal brain. The brain section images were taken at ×5 magnification and the left and right hemisphere images were taken at ×40 magnification.

At P7 of development, the left carotid artery was ligated and the mice were exposed to 10% oxygen balanced with nitrogen for 90 minutes. At 48 hours post-surgery, the brains were harvested and sectioned (Supplementary Figure 3) for immunohistochemistry to detect activation of microglia, astrogliosis and Nissl bodies. This permitted assessment of the degree of injury (categorised as severe, moderate, mild and normal) (Figs 5, 6 and 7). The results showed that reactive microglia, astrogliosis and lack of Nissl bodies were predominant in the lesioned right hemisphere of the cortex and hippocampus (Figs 5, 6 and 7). After establishing the HIE mouse model, we decided to investigate whether the use of biosensors in combination with conscious imaging would illustrate inflammation caused by the induction of the disease.

**Figure 8:**
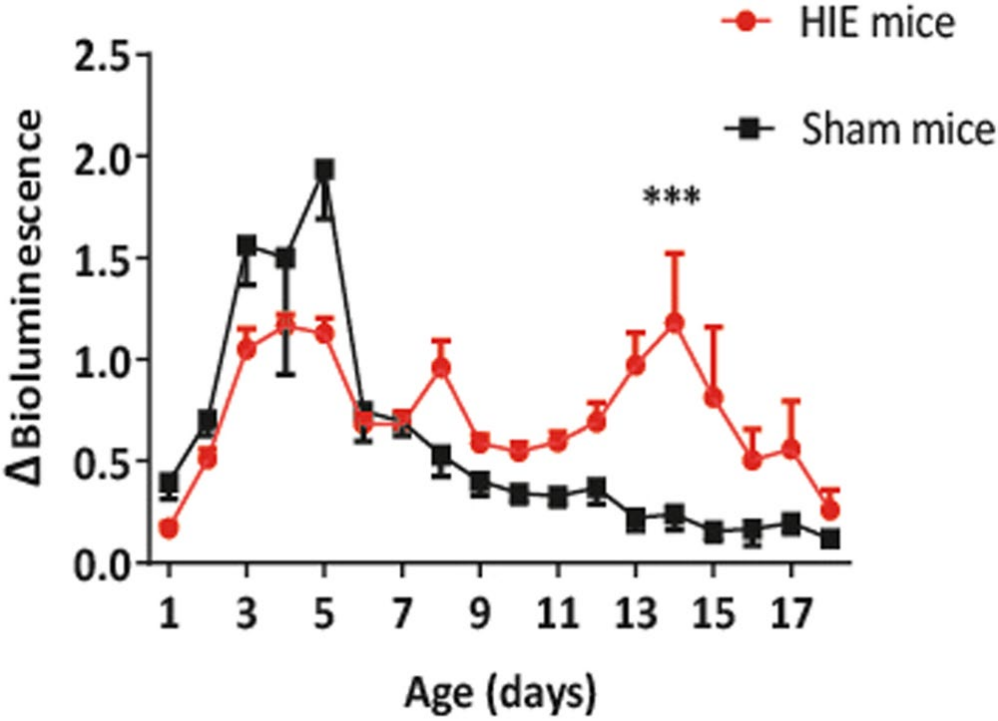
Whole-body conscious bioluminescence imaging of HIE and sham mice pre- and post-surgery. VSV-G pseudotyped NFκB biosensor was administered at birth to neonatal mice via intracranial injections (n = 22). Conscious whole-body bioluminescence imaging was conducted pre and post-surgery. The mice were split into two groups, HIE mice (n = 13) and sham control mice (n = 9). At P7 of development the HIE mice received surgery and both sets of mice were imaged daily until P19. There was a significant difference in fold change of luciferase expression between the HIE and sham mice over development (mean +/− SD, P < 0.0001). The fold change was specifically seen to be significantly higher (mean +/− SD, P < 0.001) between P14 and P16.

We therefore administered VSV-G NFκB biosensor lentivirus to newborn mice by intracranial injections (n = 22). The mice were split into two groups; HIE (n = 13) and sham (n = 9), and at P7 of development the HIE mice received surgery. Whole-body conscious bioluminescence imaging was conducted before and after surgery until developmental day 19. The results showed that there was a significant difference in fold-change of luciferase expression between the two groups of mice, P < 0.0001 (Fig. 8). A significant increase in fold-change of luciferase expression was monitored within the HIE group between P14 and P16 P < 0.001 (Fig. 8).

## Discussion

Somatic transgenic rodents have been generated previously by lentivirus vector administration to adult^25^ and neonatal mice^6^. In both studies, the luciferase transgene expression was measured in anesthetised animals. However, numerous studies provide evidence of detrimental effects of isoflurane on developing mouse tissues, particularly the brain^8, 13^. We have been able to show for the first time that luciferase expression, under the transcriptional control of a constitutive viral promoter, can be quantitated in conscious, freely moving somatic transgenic mice by whole-body bioluminescence imaging. We have demonstrated that this is feasible in dorsally situated organs (brain), and visceral organs (liver).

Additionally, we report, that neonatal intracranial injections of the two pseudotypes VSV-G and gp64 target cells of a different morphology within CNS; VSV-G specifically targets neuronal cells and gp64 targets astrocytic cells. These findings enabled us to target different subsets of cells within the CNS to monitor specific signalling pathways. This is the first time in which it has been shown that developing mice can undergo whole-body bioluminescence imaging without anaesthetics and multiple signalling pathways can be monitored within the developing brain and liver. We also report a positive correlation of brain *in vivo* luciferase expression with *ex vivo* luciferase expression. These findings rule out the ossification of the skull reducing the luciferase expression over development. Therefore, the observed reduction in luciferase reporter expression with the use of VSV-G SFFV biosensor, may possibly be due to a silencing of the lentiviral vector. A previous report has shown that lentiviral vectors containing an SFFV or CMV promoter are prone to transcriptional silencing^26^.

Our results using VSV-G NFκB biosensor are consistent with those previously shown with the use of a VSV-G NFκB biosensor within the mouse liver, lung and brain^6^. Interestingly, following administration of STAT3 biosensors we observed an upregulation of luciferase expression specifically at P10 of development. These results are consistent with previous reports that the hormone leptin is responsible for the upregulation of the JAK-STAT pathway within the hypothalamic neurons and this spikes at around P10 of development in mice^23^. Other studies have shown that in rodent pups at P16, leptin stimulated phosphorylated STAT3 expression within dopaminergic neurons^27^. We also found a significant increase in STAT3 expression in astrocytic cells by using gp64 pseudotyped lentivirus; these findings correlate with studies which have shown that hypothalamic astrocytes also express leptin receptors and therefore regulate the JAK-STAT pathway^28^.

Our data suggests that the STAT3 biosensor may be useful in studying other disease models. STAT3 signalling has shown to be vital in diffusing white matter injury within neonatal brain injury, as deleting expression of STAT3 in astrocytes increased the white matter injury and prevented the myelination maturation of oligodendrocytes^29^. A contrasting study, however, showed that preventing the expression of STAT3 in neurons specifically exerted a protective effect on neonatal hypoxic ischemic brain injury^30^.

Neonatal rodents are frequently used for models of cerebral palsy, where exposure to hypoxic conditions in combination with carotid artery ligation causes significant white matter damage^17^. We have been exploiting biosensors in a mouse model of neonatal HIE and have been able to obtain daily whole-body bioluminescence imaging measurements post-injury in conscious, freely moving pups. Establishing the mouse neonatal HIE model within outbred CD1 strain complimented previous work, which showed that CD1 mice were the most tolerant strain to hypoxic-ischaemic insult^31^. With the use of the VSV-G NFκB biosensor we were able to show a significant difference in the fold-change of luciferase expression between the HIE and sham control mice. This significant difference in expression was specifically observed 7 days post-surgery. During HIE injury there are two phases of energy failure; primary and secondary. Primary energy failure involves a depletion in glucose and oxygen which results in a decrease in ATP concentration and eventually cellular death^32^. Secondary energy failure, which occurs between 6 to 48 hours post injury^32^, is associated with the loss of high energy phosphates and the accumulation of reactive oxygen species which consequently results in another wave of apoptosis^33^. The combination of both phases of energy failures may be a cause of the significant fold-change difference between the HIE and sham control mice. As studies have shown that isoflurane exposure reduces axonal growth of primary neurons^8^, anaesthetics were avoided for whole-body bioluminescence imaging of both HIE and sham mice and therefore conscious imaging was conducted pre- and post-surgery.

Here we provide evidence that surgically induced inflammation can be monitored in a non-invasive manner and therefore employs the 3 R’s of biomedical animal research. This enables us to investigate other signalling pathways within this disease model using lentiviral biosensors. It is conceivable that this technology may also be used in combination with other bioluminescent imaging technologies. For example, bioluminescent bacteria have been used to track colonisation of the rodent intestine by *Citrobacter rodentium* ^34^ and the nasopharynx by *Streptococcus pyogenes* ^35^.

The principles of replacement, reduction and refinement are now recognised as fundamental to good standards of ethical biomedical research^36^. It is apparent that improvements in imaging technologies have not necessarily improved animal welfare in terms of anaesthesia^37^. Here we present evidence, using lentivirus vectors to generate tissue-specific somatic transgenic mice, to obtain high quality data from conscious, unrestrained animals from the day of birth until adulthood. This will not only improve the welfare of such animals, but also will likely lead to more accurate physiology and disease modelling, free of effects introduced by anaesthesia and disturbance of tissue oxygenation.

## Materials and Methods

### Lentiviral vector production and titering

The construction of the lentivirus vector payload plasmids has been previously described in Buckley *et al*.^6^.

HEK293T cells for viral production were seeded overnight in a T175 cm^2^ flask at 2 × 10^7^ cells. Cells were transfected with 50 µg of payload plasmid, 17.5 µg of pMD.G2 (VSV-G envelope plasmids) and 32.5 µg of pCMVΔR8.74 (packaging plasmid) with 1 µl of polyethylenimine (10 mM) (Sigma-Aldrich, Dorset, UK) in 12 mls of Optimem for 3 hours. Media was replaced with fresh DMEM supplemented with 10% fetal calf serum. 48 hours after transfection, vector supernatant was filter sterilized (0.22 µm) and concentrated by centrifugation at 5000 rpm for 20 hours. The centrifuged viral vector pellet was resuspended with Optimem and stored at −80 °C. All viruses were titred using a p24 Assay (Zeptometrix, Buffalo, NY, USA) following manufacturers’ protocols.

### Animal procedures

Outbred CD1 mice used in this study were supplied by Charles Rivers. All animal experiments conducted within this study were in agreement with the United Kingdom Home Office guidelines, approved by the ethical review committee and following the institutional guidelines at University College London.

### Neonatal Intracranial and Intravenous injections

For intracranial injections, mice were subjected to brief hypothermic anaesthesia on the day of birth, followed by unilateral injections of concentrated lentiviral vector (1 × 10^9^ viral particles/mL; 5 µl in total) into the cerebral lateral ventricles using a 33 gauge Hamilton needle (Fisher Scientific, Loughborough), following co-ordinates provided by Kim *et al*.^38^. For intravenous injections, pups were subjected to brief hypothermic anaesthesia followed by intravenous injections of lentiviral vectors into the superficial temporal vein, with a total volume of 20 µl of ~1 × 10^9^ viral particles/mL, using a 33 gauge Hamilton needle (Fisher Scientific). The mice were then allowed to return to normal temperature before placing them back with the dam.

### Whole-body bioluminescence imaging

Where appropriate, mice were anaesthetised with isoflurane with either 100% oxygen or medical air (21% oxygen) (Abbotts Laboratories, London, UK) and/or received an intraperitoneal injection of 15 mg/mL of D-luciferin (Gold Biotechnologies, ST Louis, USA). Mice were imaged after 5 minutes using a cooled charged-coupled device camera, (IVIS machine, Perkin Elmer, Coventry, UK) for between 1 second and 5 minutes. The regions of interest (ROI) were measured using Living Image Software (Perkin Elmer) and expressed as photons per second per centimetre squared per steradian (photons/second/cm^2^/sr).

The imaging chambers used to image conscious mice for the intracranial injected mice had the following dimensions; 5 cm × 5 cm × 6 cm. The Perspex box which was used to image the conscious mice which had received an intravenous injection is shown in Fig. 2.

### Rice-Vanucci HIE model

P7 pups were anaesthetised using isoflurane and the left carotid artery was ligated as previously described^17^. The incision was closed using tissue surgical glue (Tissue adhesive, 3 M Vetbond, USA) and the mice were returned to the nest for recovery from the surgery for 1 hour. The sham controls only received an incision at the neck and closure using surgical glue. The mice which had received artery ligation were then subjected to 10% oxygen mixed with nitrogen for up to 90 minutes, after which they were exposed to medical air (21% oxygen) for 10 minutes. The mice were returned to the dams and monitored daily.

### Collection of brain tissues

Mice were anaesthetised using Isoflurane. Their right atrium was incised and PBS (Invitrogen, Manchester, UK) was injected into the left ventricle. The brain tissue was stored in 4% PFA (Sigma-Aldrich) for 24 hours. The skull was removed and the brain tissue was fixed in 4% PFA for another 24 hours. The tissues were stored in 30% sucrose (Sigma-Aldrich) at 4 °C, until sectioned using a microtome (Carl Zeiss, Welwyn Garden City, UK) to generate 40 µm transverse sections.

### *Ex vivo* Luminometry

Brain tissue samples were lysed by adding homogenisation in 500 μl of 1× Lysis buffer (Promega). The homogenates were centrifuged for 10 minutes at 13,400 rpm (centrifuge radius, 5 cm) and the protein released from the lysis step was collected. Each sample was loaded on to a white 96 well plate. 1.5 mM of luciferin (Promega), 10 µl volume was added at a 1:1 ratio to the sample. Luminescence was measured using a FluoStar Omega microplate reader (BMG labtech) and quantified using MARS data analysis data software (BMG labtech).

### Protein assay

Protein was quantified using the Pierce BCA Protein Assay Kit (Thermo-Scientific) as per manufacturer’s protocol. Samples were loaded alongside BSA standards into a 96 well plate. 200 μl of WR reagent (1 part BCA reagent B and 50 part BCA reagent A) was added to each sample and the standards. The samples were incubated at 37 °C for 30 minutes, before being placed in a FluoStar Omega microplate reader where the sample readings were taken at 562 nm. The results were analysed in MARS data analysis data software.

### Immunoperoxidase immunohistochemistry

To visualise CD68 and GFAP immunoreactivity, sections were treated with 30% H_2_O_2_ in TBS for 30 minutes. They were blocked with 15% rabbit serum for CD68 (Vector Laboratories, Cambridge, UK) and goat serum for GFAP (Vector Laboratories) in TBST for 30 minutes. This was followed by the addition of primary antibody, rat anti-mouse CD68 (1:100; Biorad, Hertfordshire, UK), mouse anti-GFAP (1:1000; Merck Millipore, Hertfordshire, UK) in 10% serum and TBST and left on a gentle shaker over night at 4 °C. The following day the sections were treated with the secondary rat anti-rabbit antibody for CD68 (1:1000; Vector Laboratories) and goat anti-mouse (1:1000 dilution; Vector Laboratories) in 10% rat or goat serum in TBST for 2 hours. The sections were incubated for a further 2 hours with Vectastain ABC (Vector Laboratories). 0.05% of 3,3′-diaminobenzidine (DAB) was added and left for two minutes. Sections were transferred to ice cold TBS. Individual brain sections were mounted on to chrome gelatine-coated Superforst-plus slides (VWR, Poole, UK) and left to dry for 24 hours. The slides were dehydrated in 100% ethanol and placed in Histoclear (National Diagnostics, Yorkshire, UK) for 5 minutes before adding a coverslip with DPX mounting medium (VWR).

### Fluorescence immunohistochemistry

A similar protocol was followed as with the immunoperoxidase stain; however H_2_O_2_ treatment was omitted. Chicken anti-GFP (Aves labs, Oregon, USA; 1:500) was added to either of the following, mouse anti-GFAP (1:500; Millipore), and mouse anti-NeuN (1:500; Millipore). The following day the sections were incubated with biotinylated secondary antibodies (goat anti-mouse) all at 1:1000 dilutions for 2 hours. Sections were incubated with a fluorescent secondary antibody for 2 hours; goat anti-chicken Alexa Fluor 488 (1:200; Invitrogen) and goat anti-mouse Alexa Fluor 568 (1:500; Invitrogen). The sections were treated with DAPI (5 mg/mL) in the dark for a couple of minutes. They were transferred to ice cold TBS. Each individual brain section was mounted on to slides and left to dry for a couple of hours. Once dried, a cover slip was added using Fluromount G (Ebioscience, Hatfield, UK).

### Nissl

Transverse brain sections were mounted onto chrome gelatine-coated Superforst-plus slides and were left at room temperature for 24 hours. The slides were transferred to 70% ethanol for 5 minutes. After which they were transferred to the Nissl solution, which was produced by adding 4 g of Cresly Violet (VWR) to 10% ethanol, heated and filtered. After which the slides underwent a series of 2-minute incubations in the following solutions; dH_2_0 × 2, 70% ethanol, 90% ethanol, 96% ethanol, 96% ethanol 1% acetic acid (Sigma), 100% ethanol, 100% isopropanol and xylene (VWR) × 3. The slides were then coverslip with DPX mounting medium.

### Statistics

For the anesthetised and un-anesthetised comparison of luciferase expression (Figs 1 and 2), a Mann-Whitney test was carried out. Spearman’s rank correlation was carried out for Fig. 1B. Fold change of bioluminescence (Figs 4 and 5) was obtained by expressing photon counts as a ratio over the median of luciferase expression from days 1 to 5 (Fig. 4) and 3 to 6 (Fig. 5). Median was used, as it is a much more robust measure of central tendency. Two-way ANOVA’s and a multiple comparison undertaken in Figs 4 and 5.

## Electronic supplementary material

Supplementary Information
