# Supplementary material for: Continual conscious bioluminescent imaging in freely moving somatotransgenic mice
Source: Sci Rep. 2017 Jul 25;7:6374. doi: 10.1038/s41598-017-06696-w (PMC5526882; doi:10.1038/s41598-017-06696-w)
Supplement: Supplementary file 1 — Supplementary Information [file 41598_2017_6696_MOESM1_ESM.pdf]

Continual conscious bioluminescent imaging in freely moving somatotransgenic mice

Rajvinder Karda[1,4], Dany P. Perocheau[1], Natalie Suff[1], Joanne Ng[1], Juliette M.K.M. Delhove[2], Suzanne M.K. Buckley[1], Samantha Richards[1], John R. Counsell[2], Henrik Hagberg[3], Mark R. Johnson[4], Tristan R. McKay[5] & Simon N. Waddington[1]

1. Gene Transfer Technology Group, Institute for Women's Health, University College London, UK
2. Institute of Child Health, University College London, UK
3. Department of Perinatal Imaging & Health, King's College London, UK
4. Department of Surgery and Cancer, Imperial College London, UK
5. Centre for Biomedicine, Manchester Metropolitan University, Manchester, UK

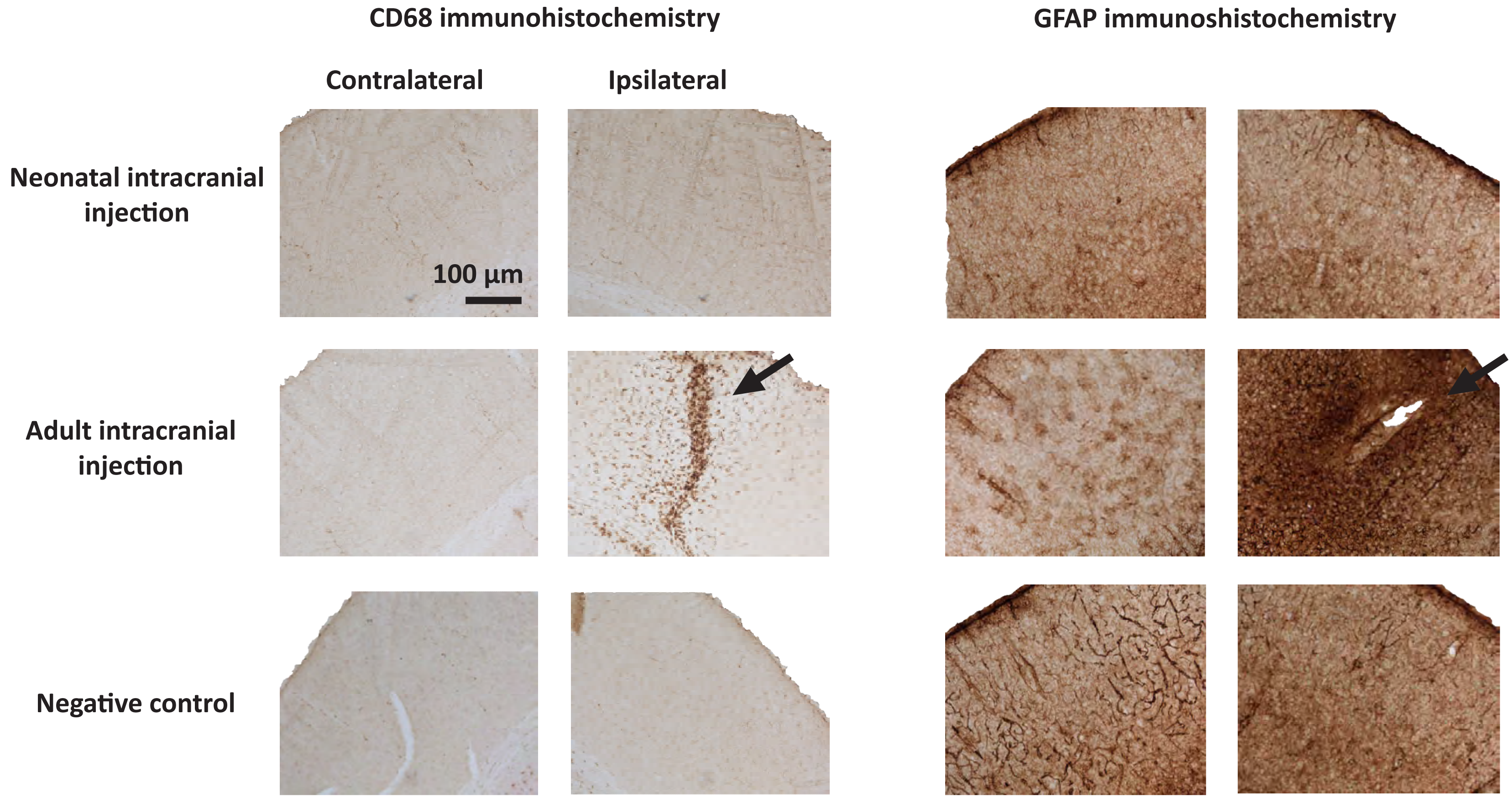

Supplementary figure 1 - GFAP and CD68 immunohistochemistry showing no scarring after neonatal intracranial injections

Immunohistochemistry to detected GFAP and CD68 was conducted on adult brains (P35) from mice which had received the VSV-G SFFV biosensor via intracranial injection at birth. This revealed no signs of astrogliosis or reactive microglia in these brain sections, shown in the neonatal intracranial panel. Injected brains were indistinguishable from the negative control, uninjected mice. However, astrogliosis and reactive microglia were observed within the positive controls from adult mice following intracranial injection at P21 of development. The scarring is shown by the black arrows in both CD68 and GFAP immunohistochemistry panels. Images were taken at x40 magnification.

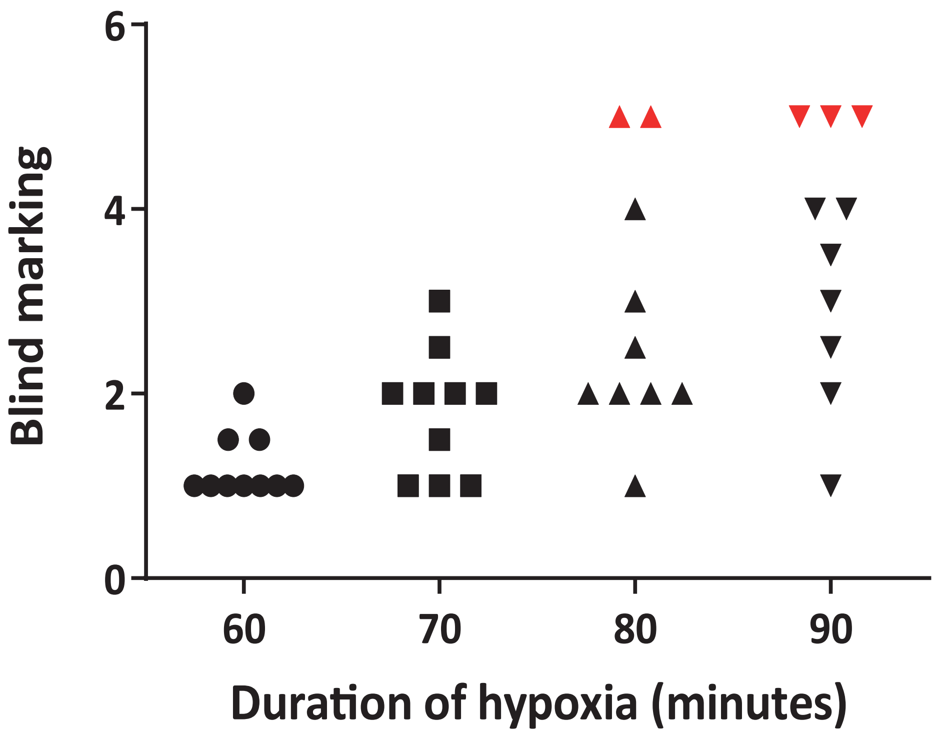

Supplementary figure 2 – CD1 HIE mice exposed to different duration of hypoxia.

P7 CD1 mice underwent surgery, where their left carotid artery was ligated. The mice were then exposed to different time points of hypoxia, 60, 70, 80 and 90 minutes (n=10 for each time point). 48 hours post-surgery the brains were blind marked for injury, 1 as normal, 2 mild, 3 moderate, 4 severe, 5 death of mice (shown in red). Exposure to 90 minutes revealed a variable injury score.

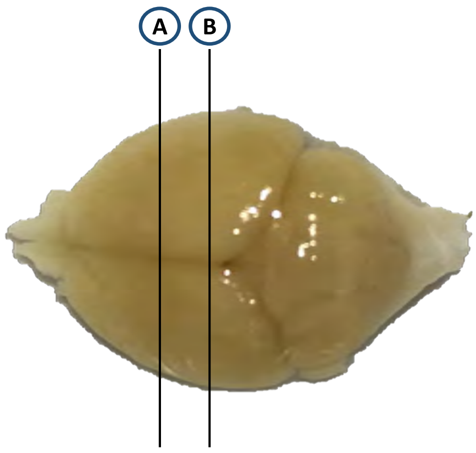

Supplementary figure 3 – Whole brain showing sections which underwent immunohistochemistry for CD68 and GFAP.

The cortex shown by line A and hippocampus shown by B underwent immunohistochemistry, as these regions are prone to damage by hypoxic-ischaemic insult [22].
